# Digital spatial profiling identifies phospho-JNK as a biomarker for early risk stratification of aggressive prostate cancer

**DOI:** 10.3389/fonc.2025.1572299

**Published:** 2025-06-05

**Authors:** Samaneh Eickelschulte, Adam Kaczorowski, Florian Janke, Anja Lisa Riediger, Olga Lazareva, Sarah Böning, Glen Kristiansen, Constantin Schwab, Albrecht Stenzinger, Holger Sültmann, Stefan Duensing, Anette Duensing, Magdalena Görtz

**Affiliations:** ^1^ Junior Clinical Cooperation Unit, Multiparametric Methods for Early Detection of Prostate Cancer, German Cancer Research Center (DKFZ), Heidelberg, Germany; ^2^ Department of Urology, Heidelberg University Hospital, Heidelberg, Germany; ^3^ Division of Cancer Genome Research, German Cancer Research Center (DKFZ), National Center for Tumor Diseases (NCT), Heidelberg, Germany; ^4^ Molecular Urooncology, Department of Urology, Heidelberg University Hospital, Heidelberg, Germany; ^5^ Faculty of Biosciences, Heidelberg University, Heidelberg, Germany; ^6^ Division of Computational Genomics and Systems Genetics, German Cancer Research Center (DKFZ), Heidelberg, Germany; ^7^ Institute of Pathology, University Hospital Bonn, Bonn, Germany; ^8^ Institute of Pathology, Heidelberg University, Heidelberg, Germany; ^9^ Institute of Pathology, Tissue Bank of the National Center for Tumor Diseases (NCT), Heidelberg, Germany; ^10^ German Cancer Consortium (DKTK), Heidelberg, Germany; ^11^ Precision Oncology of Urological Malignancies, Department of Urology, Heidelberg University Hospital, Heidelberg, Germany

**Keywords:** prostate cancer, digital spatial profiling (DSP), p-JNK, risk stratification, biomarker discovery

## Abstract

**Background:**

Prostate cancer (PCa) is a highly heterogeneous disease, ranging from indolent to highly aggressive forms. Ongoing research focuses on identifying new biomarkers to improve early risk stratification in PCa, addressing current limitations to accurately evaluate disease progression. A promising new approach to aid PCa risk stratification is digital spatial profiling (DSP) of PCa tissue.

**Methods:**

A total of 94 regions of interest from 38 PCa patients at first diagnosis were analyzed for the expression of 44 proteins, including components of the PI3K/AKT, MAPK, and cell death signaling pathways as well as immune cell markers. An additional validation cohort consisting of 154 PCa patients with long-term follow-up data was analyzed using immunohistochemistry (IHC) to assess the consistency of the identified biomarkers across a larger sample set.

**Results:**

DSP identified the proliferation marker Ki-67 and phosphorylated c-Jun N-terminal protein kinase T183/Y185 (p-JNK), a member of the MAPK signaling pathway, as significantly upregulated proteins in aggressive PCa (Gleason grades 4 or 5) compared to indolent disease (Gleason grade 3; *p*<0.05). The upregulation of p-JNK was confirmed by IHC. High p-JNK expression was associated with a shorter time to biochemical recurrence (log-rank, *p*=0.1).

**Conclusion:**

Our results indicate that p-JNK may contribute to PCa progression and serve as an early biomarker for aggressive PCa stratification. Identification of this biomarker through DSP could prove crucial in advancing disease management and addressing the critical unmet need for more targeted therapies in the treatment of PCa. Further studies are warranted to evaluate the role of p-JNK in PCa progression.

## 1 Introduction

Prostate cancer (PCa) is the most frequent non-cutaneous malignancy in men (1) and is characterized by its high heterogeneity and complexity. In case of elevated prostate-specific antigen (PSA) and/or suspicious digital rectal examination, a prostate biopsy is performed and histopathologically evaluated using the Gleason grading system. The Gleason score is based on the classification of prostate adenocarcinoma growth patterns, with the most common and most aggressive Gleason grades defining the score in prostate biopsy specimens, whereas the two most common Gleason grades define the score in prostatectomy specimens (2, 3). The distinction between Gleason pattern 3 and 4 is crucial, as pattern 4 indicates a shift towards more aggressive disease (4).

The Gleason grading system has consistently demonstrated significant prognostic value in stratifying PCa. However, recent research has highlighted the heterogeneity of molecular alterations, posing challenges for its diagnosis and treatment (5, 6). Therefore, additional diagnostic methods are often necessary to effectively stratify PCa into high- and low-risk categories, enabling the selection of appropriate therapies for each patient at the time of initial diagnosis. For patients with indolent disease, active surveillance with regular monitoring is the preferred approach, as it avoids unnecessary radical treatment while ensuring timely intervention if the disease progresses (7).

This heterogeneity in PCa is further emphasized by spatial relationships between cell populations, which can significantly influence disease progression and treatment outcomes (8). Consequently, spatial profiling of protein expression might aid in better understanding the biology of PCa and facilitate risk stratification. Biomarkers related to immune cell signaling and cell death pathways hold significant potential for improving PCa prognosis (9–11). Especially, expression of genes related to cell cycle progression, in particular, Ki-67 have been associated with poor prognosis across various types of cancer (12–14), including PCa (15). Additionally, the PI3K/AKT pathway, regulating cell growth, survival, and metabolism, provides valuable insights into the molecular mechanisms that drive aggressive PCa (16). Recent research has also revealed that the complex interactions between the mitogen-activated protein kinase (MAPK) signaling pathway and other cell-signaling cascades can further contribute to PCa progression, highlighting its role in the disease’s development (17). However, the full extent of the MAPK signaling network during prostate tumorigenesis, as well as the involvement of spatial niches in tumor progression and disease recurrence, remain to be fully determined. Studies have shown that c-Jun N-terminal kinase (JNK), a key member of the MAPK signaling pathway, is associated with tumor progression and survival in various cancers (18–20) and contributes to the growth of PCa (21). In this proof-of-concept study, we used digital spatial profiling (DSP) to examine protein expression across four key cellular pathways involved in PCa. We identified Ki-67 and phospho-JNK T183/Y185 (p-JNK) as key drivers of disease progression and recurrence distinguishing aggressive Gleason patterns 4 and 5 PCa from indolent Gleason pattern 3 disease.

## 2 Methods

### 2.1 Study population

Tissue microarrays (TMAs) were obtained from localized and locally advanced (N1M0) PCa patients who had undergone radical prostatectomy at first diagnosis at the Department of Urology, University Hospital of Heidelberg, Germany. The study design comprised specimens from 192 patients, divided into two independent cohorts: one cohort subjected to analysis by DSP (n=38) and a larger validation cohort with long-term follow-up data (n=154) analyzed by immunohistochemistry (IHC) (**Table 1**). All tissue-based experiments in this study were in accordance with the regulations of the tissue bank as well as under the approval of the Ethics Committee of the Medical Faculty of the University of Heidelberg (votes: S-130/2021 and S-864/2019). Written informed consent was obtained from all participants.

**Table 1:** Patient characteristics.

Prostate cancer patients	DSP cohort (N=38)	IHC cohort (N=154)
Age, years, median (range)	65 (49–76)	66 (48-79)
PSA level, ng/ml, median (range)^1^	7 (1.52–40)	0.36 (0.18-1165)
ISUP grade group, Gleason score, n (%)^2^		
2 (7a)	27 (71)	38 (25)
3 (7b)	4 (11)	51 (33)
4 (8)	0 (0)	60 (39)
5 (9-10)	7 (18)	4 (3)
pT stage, n (%)		
T2	22 (58)	26 (17)
T3 T4	16 (42) 0 (0)	114 (74) 14 (9)
pN stage, n (%)		
N0	25 (66)	44 (29)
N1	8 (21)	110 (71)
Nx	5 (13)	0 (0)
cM stage, n (%)		
M0	36 (95)	149 (96)
M1 Mx	2 (5) 0 (0)	4 (3) 1 (1)
R status		
R0	21 (55)	49 (32)
R1	16 (42)	67 (43)
Rx	1 (3)	38 (25)

^1^Data available for 84/154 patients from IHC cohort.

^2^Data available for 153/154 patients from IHC cohort.

### 2.2 TMA construction

TMAs were constructed using 1 mm diameter cores punched from each formalin-fixed paraffin-embedded (FFPE) tissue blocks. TMAs for both DSP and IHC cohorts were retrieved from the tissue bank of the National Center for Tumor Diseases Heidelberg. The IHC cohort primarily comprised patients with high-risk PCa (11, 22). For each patient, we separated tissues into Gleason 3 and Gleason 4 patterns, and, when available, also collected tissues from those with Gleason 5.

### 2.3 Digital spatial profiling sample preparation

The GeoMx^®^ DSP platform (NanoString Technologies, Seattle, Bruker Corporations, Billerica, MA, USA) was used for the spatial analysis of PCa TMAs, and the TMA cores were prepared as described previously (8, 23). Briefly, slides were deparaffinized in staining jars by incubation for 3 x 5 min in xylene followed by rehydration for 2 x 5 min in 100% ethanol, 2 x 5 min in 95% ethanol and 2 x 5 min in deionized water. Antigen retrieval was performed in 1X citrate buffer (pH 6) for 15 min in a pressure cooker at high temperature and high pressure. The slides were then washed in 1X TBS-T buffer, and the tissue sections were blocked with Buffer W for 1 h in a humidity chamber at room temperature (RT) before incubation with a mixture of the barcoded antibodies and morphology markers overnight at 4°C. In this study, the NanoString barcoded antibody panels consisted of the human protein Immune cell profiling, PI3K/AKT and MAPK signaling, and cell death modules (**Supplementary Table 1**). CD45 was used to identify T-cells, panCK to label epithelial cells, and SYTO13 to stain cell nuclei, serving as morphological markers for the visualization of the tissue architecture using the NanoString Solid Tumor TME Morphology Kit. After antibody staining, slides were washed 3 × 10 min in 1X TBS-T and post-fixed in 4% paraformaldehyde at RT for 30 min, followed by 2 × 5 min washes in 1X TBS-T. Nuclei were stained with 500 nM SYTO13 for 15 min at RT and rinsed with 1X T-TBS before loading into a GeoMx^®^ instrument (v.2.4.2.2).

### 2.4 Digital spatial profiling analysis

Slides were scanned with the GeoMx instrument and regions of interest (ROIs) were carefully selected by an experienced pathologist (S.D.) using morphology markers, as described previously (23). To aid in selecting ROIs, consecutive hematoxylin- and eosin-stained (H&E) sections were examined simultaneously under a microscope. The selection process focused on capturing cancerous tissue while excluding non-cancerous regions. Within each TMA core, ROIs were carefully chosen to ensure that only carcinoma areas were included. No further segmentation was performed within the selected ROIs. Each ROI was illuminated with UV light and cleaved barcodes were collected and hybridized with fluorescent probes for 16 h at 67°C. Hybridized probes were then processed using the nCounter^®^ MAX/FLEX Prep Station (v4.1.0.1) and counted using the nCounter^®^ Digital Analyzer (v4.0.0.3). Data analysis was conducted using the GeoMx^®^ DSP analysis suite and R (v.4.2.3). Raw counts generated from the DSP were normalized using the housekeeping proteins GAPDH and S6 to ensure consistency (**Supplementary Table 2**), followed by log_2_ transformation to minimize variability. Quality control measures were rigorously applied, and samples with inadequate signal-to-noise ratios were excluded from further analysis. Visualization of the data was achieved using R (v.4.2.3).

### 2.5 Immunohistochemical staining

TMA cores were stained for p-JNK expression and prepared as described previously (23). Heat-mediated antigen retrieval was performed using antigen retrieval solution (Dako, Glostrup, Denmark) and slides were blocked in goat serum. Anti-p-JNK1/2 (T183/Y185, Invitrogen/ThermoFisher Scientific, Waltham, MA, USA) was used as a primary antibody. Biotinylated anti-rabbit secondary antibody (ab97049, 1:200; Abcam) and streptavidin-peroxidase conjugate (1:1250, Merck/Sigma-Aldrich, Taufkirchen, Germany) were used to detect the primary antibody. For tissue staining and counterstaining, 3,3’-Diaminobenzidine (Abcam, Cambridge, UK) and Hematoxylin Gill I (Sigma Aldrich, St. Louis, MO, USA) were used. TMA cores were then dehydrated, and mounted with HistoMount solution (Life Technologies, Carlsbad, CA, USA). Single TMA cores were stained for p-JNK expression, according to the described immunohistochemistry protocol. p-JNK expression was assessed by a semiquantitative immunoreactive score (IRS) considering signal intensity and proportion of positively stained cells.

### 2.6 Statistical analysis and R packages

Statistical analysis of spatial protein expression data was performed using the GeoMx^®^ DSP software (v2.4.2.2) and R (v.4.2.3). To identify statistically significant differences in protein expression between Gleason grades 4/5 and Gleason grade 3, we conducted Mann–Whitney U tests on log_2_-transformed protein expression data across the DSP cohort. *P* values were adjusted using the Benjamini-Hochberg method, with a significance threshold set at *p* < 0.1. Survival data were evaluated according to Kaplan–Meier and survival between groups was compared using the log-rank test (*p* < 0.1). R packages used for data visualization were ggplot2 (3.5.1), pheatmap (1.0.12) and survminer (0.4.9).

## 3 Results

### 3.1 DSP of protein expression in PCa patients

**Figure 1:**
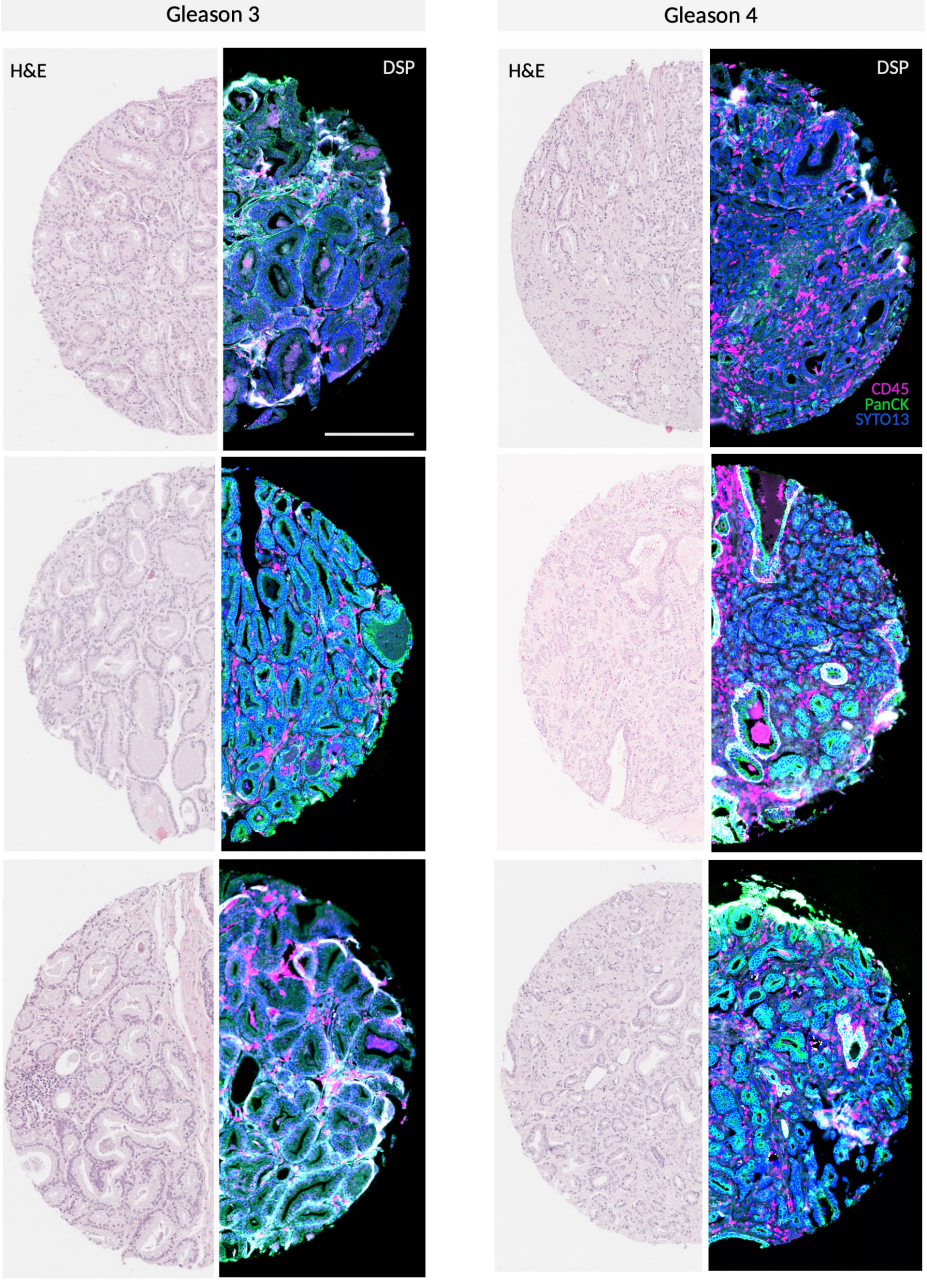
Representative GeoMx^®^ DSP scan of PCa specimen Gleason 3 and 4 TMA cores stained with morphology markers SYTO13, CD45 and PanCK along with H&E staining. Scale bar = 250 µm.

**Figure 2:**
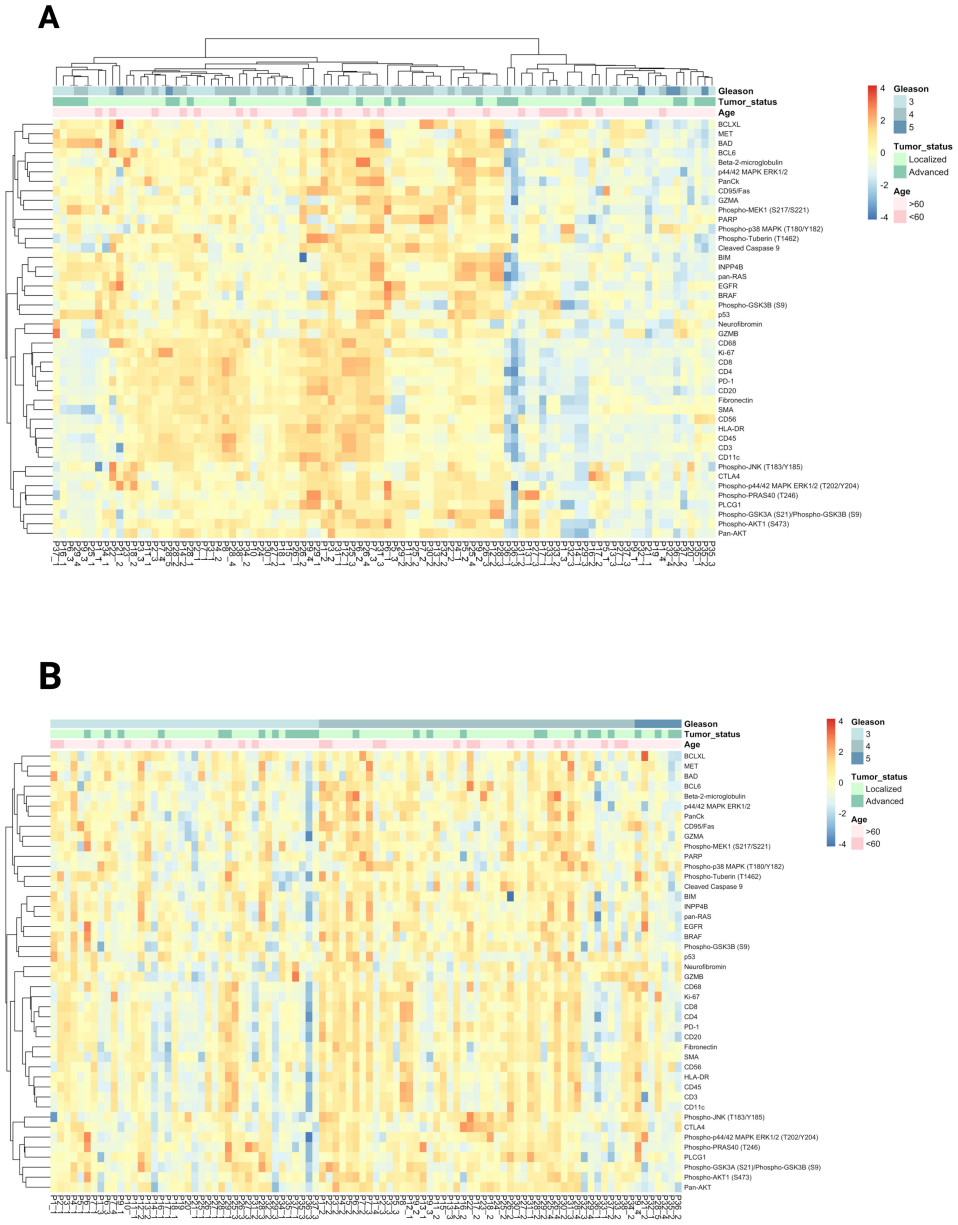
**(A)** Unsupervised clustering and heatmap of DSP protein expression data obtained from 94 Gleason grade ROIs from 38 PCa patients. Data normalized to house keeper GAPDH & S6. **(B)** Heatmap visualization of target protein expression across patient samples ordered by Gleason score.

To characterize the spatial distribution of tumor cells in PCa, we constructed TMAs using samples from various sites within the prostatectomy specimen of 38 PCa patients (**Figure 1**). These samples were selected to represent tumor heterogeneity, incorporating regions with different Gleason grades. We selected and analyzed a total of 94 ROIs (**Supplementary Table 3**) to profile the expression of 44 proteins, including key components of the PI3K/AKT, MAPK, and cell death signaling pathways as well as immune cell markers. Due to differences in staining quality and tissue characteristics, we were able to analyze ROIs containing Gleason 3 and 4 patterns from 21 patients. Two patients had ROIs with Gleason grades 3 and 5, but no Gleason 4 pattern. Additionally, 11 patients had ROIs containing either Gleason 3 or 4 pattern and four patients had ROIs corresponding to all three Gleason patterns (3, 4, and 5) (**Supplementary Table 3**). An unsupervised hierarchical clustering was performed to further explore the expression patterns. This analysis revealed significant variability in protein expression and pathway activity between Gleason pattern 3 (indolent disease) and Gleason patterns 4 or 5 (aggressive PCa) as shown in **Figure 2A**. To assess the impact of the Gleason patterns, we arranged the columns (patient samples) according to increasing Gleason grades, while retaining unsupervised hierarchical clustering of the rows (target proteins). As shown in **Figure 2B**, this approach allows for the evaluation of gene expression patterns in relation to Gleason patterns.

### 3.2 DSP identifies p-JNK and Ki-67 as biomarkers for prostate tumor risk stratification

**Figure 3:**
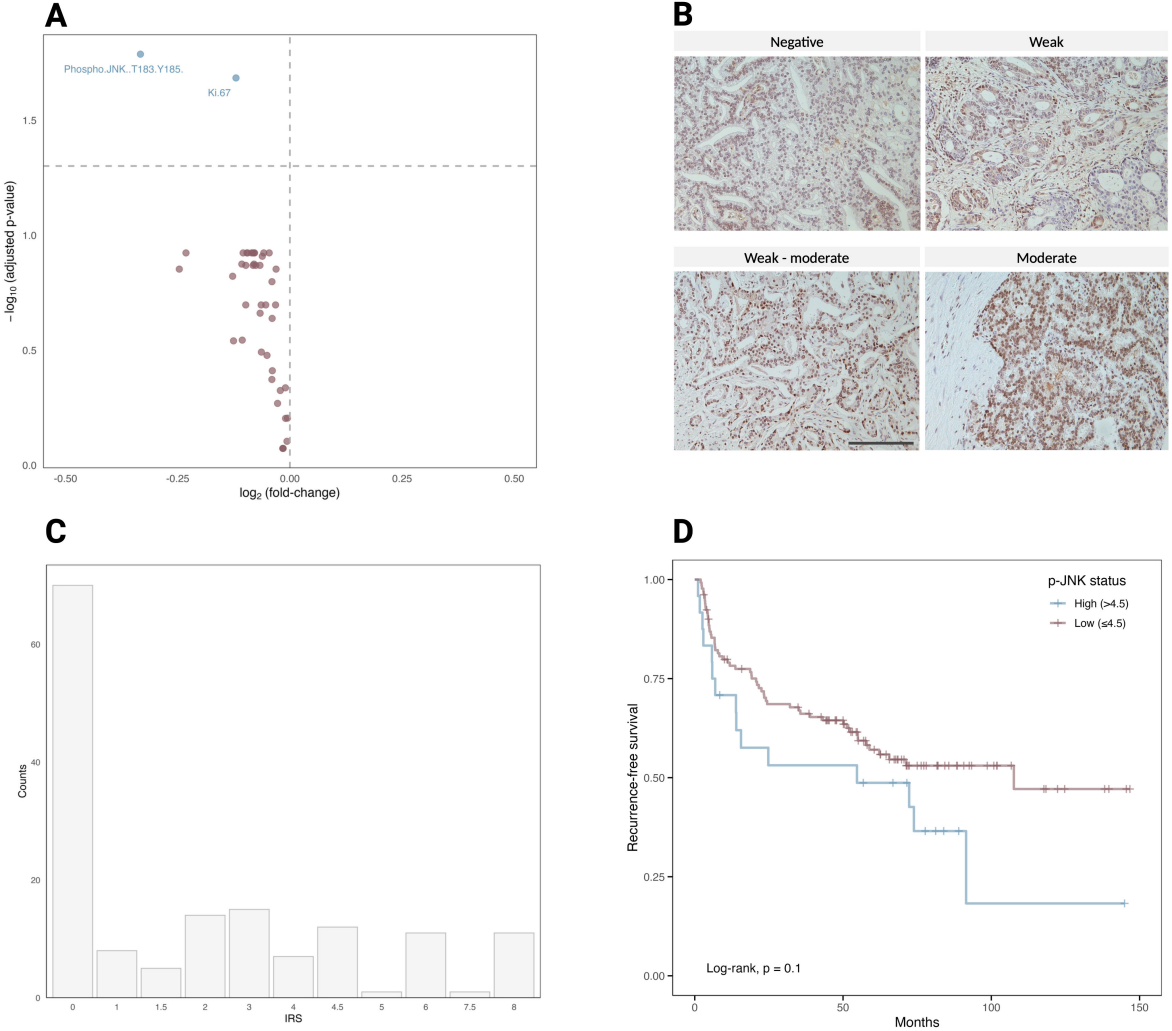
**(A)** Volcano plot of 44 differentially expressed target proteins in 94 ROIs comparing Gleason 3 to Gleason 4 and 5. The vertical dotted lines represent the log_2_(fold change) of 0.5 and − 0.5. The horizontal dotted line shows the adjusted *p*-value (Benjamini-Hochberg) < 0.1. Significantly upregulated proteins are p-JNK and Ki-67, highlighted in blue (*p* = 0.01 and 0.02, respectively). **(B)** A representative patient in the IHC cohort stained with p-JNK (Thr183/Tyr185). The staining intensity was scored on a scale of negative, weak, weak-moderate and moderate and used to calculate the IRS of p-JNK. Scale bar = 100 µm. **(C)** Histogram showing the distribution of p-JNK IRS in the validation cohort of 154 patients. **(D)** Kaplan–Meier curve for 154 patients of the validation cohort, stratified into patients with high (> 4.5; n = 24) or low (≤ 4.5; n = 130) p-JNK IRS.

The analysis of 94 ROIs derived from immune cell profiling, PI3K/AKT, MAPK signaling pathways, and cell death modules revealed the upregulation of several proteins when comparing Gleason 4 and 5 to Gleason 3 samples. A Wilcoxon rank-sum test was performed to assess the statistical significance of differences in target expression between samples with Gleason grade 3 and those with Gleason grades 4 or 5. p-JNK and Ki-67 were the only significantly upregulated targets (adjusted *p* value of 0.01 and 0.02, respectively) in Gleason 4 and 5 PCa compared to Gleason 3 PCa (**Figure 3A**). The ROIs were selected to evaluate prostate tumor cells, and p-JNK expression could be attributed to tumor cells in H&E sections. Upregulation of p-JNK was observed in the ROIs selected from TMA cores representing prostate cancer tissue, with Gleason grades 4 or 5 showing a higher expression of p-JNK compared to Gleason grade 3. p-JNK expression was found to be homogeneous within the tumor in the respective ROI.

### 3.3 p-JNK expression correlates with a shorter time to biochemical recurrence

**Figure 4:**
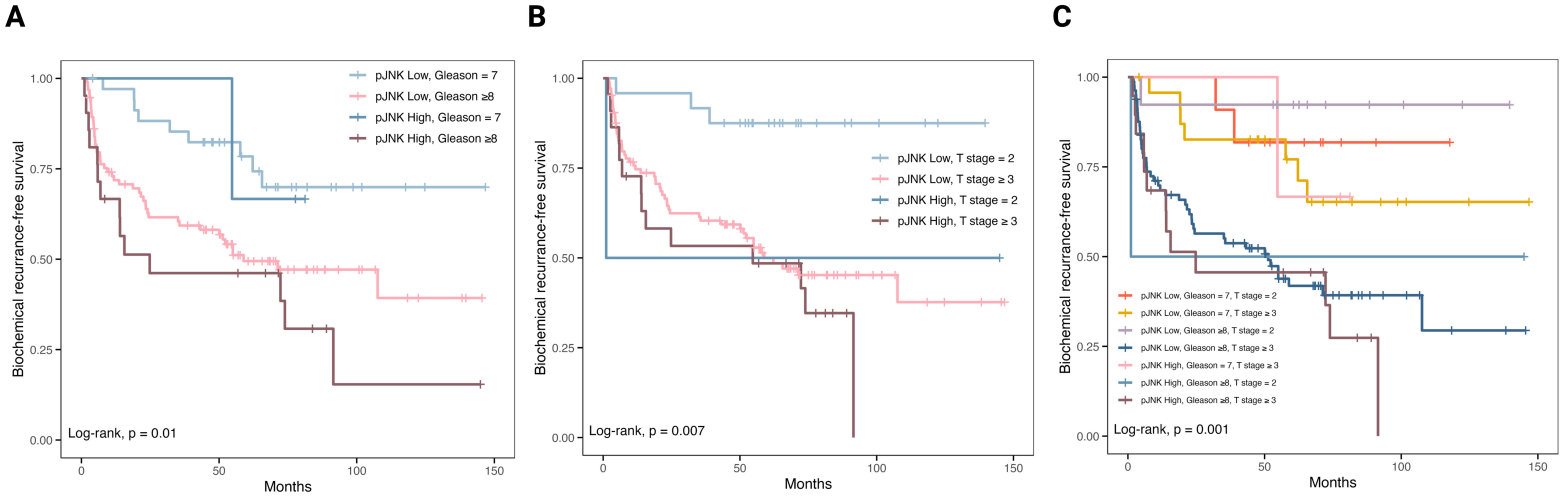
Combined analysis of p-JNK status with pathology-derived parameters. **(A)** p-JNK status combined with Gleason score. **(B)** p-JNK status combined with T stage. **(C)** p-JNK status combined with both Gleason score and T stage. p-JNK expression was stratified into high and low according to the immunoreactive score (cutoff >4.5).

To further investigate the role of JNK phosphorylation in tumor progression, we conducted a validation cohort analysis using TMAs of 154 PCa patients with long-term follow-up data (**Table 1**). The validation cohort was stained for p-JNK expression using IHC. For each TMA core, the p-JNK immunoreactive score (IRS) was calculated based on two components: the intensity of p-JNK staining and the fraction of positively stained cells. The staining intensity was scored on a scale of 0 to 2.5, where 0 represents negative, 1 indicates weak staining, 1.5 signifies weak to moderate staining, 2 denotes moderate staining and 2.5 corresponds to moderate to strong staining (**Figure 3B**). The proportion of positive cells was defined as follows: negative staining was scored as 0, below 10% of stained cells was scored as 1, 10–50% was scored as 2, 50% to 90% was scored as 3, and above 90% was scored as 4. The IRS was then calculated by multiplying the staining intensity score by the score of the proportion of positive cells. The highest IRS of all evaluable TMA cores per patient (TMA cores per patient [median, range] = 3, 1-6) was considered the IRS of an individual patient and the p-JNK IRS distribution ranged from 0 to 8 (**Figure 3C**). When patients were stratified into two groups according to p-JNK IRS, Kaplan-Meier analysis showed that patients with high p-JNK expression (p-JNK IRS > 4.5) tended to have a shorter time to biochemical recurrence (*p* = 0.1; **Figure 3D**). To evaluate the sensitivity of p-JNK as a biomarker for predicting biochemical recurrence, we determined the true positive and false negative rates in the cohort of 153 patients from the IHC dataset with available Gleason scores. True positive cases were defined as Gleason ≥8 cases correctly identified as aggressive by p-JNK, while false negative cases were Gleason ≥8 cases incorrectly classified as non-aggressive by p-JNK. For specificity, we assessed the number of Gleason 7 cases correctly identified as non-aggressive by the biomarker p-JNK (true negatives) and the cases misclassified as aggressive by p-JNK (false positives). Our results showed a sensitivity of 18.2% and a specificity of 92.1% for p-JNK. Due to the low sensitivity but high specificity, p-JNK behaves as a rule-in biomarker rather than a screening test for aggressive disease. A positive p-JNK result means high probability that patients truly harbor aggressive disease. To further explore prognostic factors for biochemical recurrence, we incorporated pathology−derived parameters (*e.g*., Gleason score, and T stage) into our analysis. Using Kaplan-Meier curves, we compared recurrence-free survival across different risk groups defined by combination of these variables. This approach allowed us to determine whether combining these pathology−derived parameters with p-JNK offers improved predictive value for biochemical recurrence (**Figure 4A-C**). Our results suggest that combining p-JNK with pathology−derived parameters, Gleason score and T stage, enhances the accuracy of predicting aggressive PCa and is associated with a more unfavorable progression-free survival in PCa (*p*<0.05 for the combination of p-JNK with Gleason score, p-JNK with T stage and p-JNK with Gleason score and T stage, respectively).

## 4 Discussion

Heterogeneous malignancies such as PCa pose significant challenges for risk stratification and clinical decision-making in patients. Addressing this challenge requires advanced diagnostic techniques to assess multiple tumor regions accurately, classify PCa subtypes, and identify biomarkers associated with aggressive disease (24). Emerging technologies such as DSP offer promising opportunities to better characterize intratumoral heterogeneity, particularly in FFPE slides, which are the standard method for storing tumor tissue (8, 25). DSP is particularly well-suited for comparing different regions within a tumor, as it enables precise selection of specific areas of interest, offering a notable advantage in spatially heterogenous tumors (11, 23, 26).

In this study, we utilized two independent cohorts of PCa patients: one cohort (n=38) to identify biomarkers for PCa aggressiveness via DSP and a larger validation cohort (n=154) with available follow-up data to correlate these markers to biochemical recurrence using IHC. Through the analysis of the spatial composition in TMA of 38 PCa patients, we demonstrated that PCa with varying Gleason grades exhibited distinct expression patterns for two key proteins, Ki-67 and p-JNK, when comparing Gleason pattern 3 to Gleason pattern 4 or 5 PCa. Considering that Gleason grade 3 tumors represent a less aggressive form of PCa, whereas Gleason grade 4 or 5 tumors are associated with more aggressive disease, the differential expression of Ki-67 and p-JNK suggests that these proteins may play a role in promoting tumor aggressiveness. Ki-67, a marker of cellular proliferation, has been repeatedly identified as a promising prognostic biomarker for PCa, with a higher Ki-67 index correlating with worse biochemical recurrence-free survival (27). JNK, a member of the MAPK family, regulates a wide range of cellular processes (28). In this study, we further focused on p-JNK because, unlike Ki-67, its role as a biomarker for PCa aggressiveness remains less established. In our validation cohort of 154 PCa patients, immunostaining with p-JNK antibody revealed that higher p-JNK expression was associated with a shorter time to biochemical recurrence, highlighting a potential role of p-JNK upregulation in tumor progression. Combination of p-JNK status and pathology-derived parameters such as Gleason score and T stage was associated with improved risk stratification and provided a more accurate prediction of progression-free survival in PCa. Given the high specificity but low sensitivity of p-JNK observed in our study, its potential clinical applications lie in refining patient stratification. Specifically, p-JNK positivity can be used to identify a subset of patients who could benefit from earlier or more aggressive adjuvant therapies such as radiotherapy or systemic treatment following prostatectomy. In cases where p-JNK is negative, it may serve a valuable role when combined with other strong negative clinical or pathology-derived predictors.

JNK has a dual role in cancer, acting either as a tumor suppressor by inducing cell death or as a promoter of cell proliferation, depending on factors such as the type of stimuli, tissue specificity, and signal intensity (29, 30). In response to stress, JNK can initiate cell death by activating pro-apoptotic transcription factors (31). Conversely, the loss of JNK signaling can contribute to tumor formation through phosphorylation of specific signaling proteins that stimulate growth-related gene expression (28). In its dual role, JNK also promotes cell survival by downregulating FoxO1-dependent autophagy (32–34). However, prolonged JNK activation often leads to apoptosis mediated by TNFα (35). In other tumor entities, JNK pathway deficiency was shown to support HER2+-driven breast cancer (36), while elevated JNK expression was associated with worse prognosis in colorectal cancer patients (37). Increasing evidence indicates that JNK signaling is closely related to cellular senescence, a state of permanent growth arrest (38). The secretory profile of senescent cells can modify the tissue microenvironment by evading immune surveillance and creating conditions that robustly drive PCa progression (39, 40).

In PCa, JNK contributes to both apoptosis and tumor progression, reflecting its complexity in cancer pathway regulation. Activation of JNK has been shown to increase sensitivity to chemotherapy and promote apoptosis in PCa cells, indicating its potential in sensitizing tumors to treatment (41). Similarly, inhibition of autophagy through the JNK pathway significantly enhances apoptosis in PC3 cells (42). However, JNK also promotes prostate tumor growth through interactions with the tumor microenvironment (40, 42). Higher JNK expression corelates with higher Gleason score and is associated with shorter overall and progression-free survival in patients with castration-resistant PCa (43). These findings suggest that targeting the JNK pathway could be a promising therapeutic strategy for PCa, potentially improving the effectiveness of current treatments. In addition, JNK as a biomarker in early PCa could help differentiate aggressive from indolent disease, thereby improving decision-making regarding active surveillance versus radical treatment in PCa.

This study has several limitations including the small DSP cohort. The use of a TMA containing Gleason 3, Gleason 4, and Gleason 5 tumor areas is likely insufficient to fully capture the spatial distribution of p-JNK expression across the entire tumor. The relatively small protein panel includes primarily oncogenes, while tumor suppressor genes were underrepresented. Furthermore, whether p-JNK is also a biomarker in patients undergoing prostate biopsy remains to be determined. Although our Kaplan-Meier analysis suggested a potential association between high p-JNK expression and shorter biochemical recurrence-free survival, this trend did not reach statistical significance (*p* = 0.1). Therefore, our findings regarding p-JNK must be considered exploratory, and further validation in larger cohorts is required to establish its prognostic value and its clinical utility beyond its initial identification as a biomarker.

In summary, differentially expressed proteins in aggressive versus indolent PCa can shed light on the molecular mechanisms driving disease progression. Proteins that are upregulated in high-grade tumors could serve as potential biomarkers and open new therapeutic avenues. This approach can ultimately contribute to the field of personalized medicine by helping clinicians to tailor treatment strategies based on the molecular characteristics of the tumor.

## Data Availability

The original contributions presented in the study are included in the article/**Supplementary Material**. Further inquiries can be directed to the corresponding authors.

## Supplementary material

Supplementary Table 1 Overview of Nanostring GeoMx^®^ DSP protein panels.

Supplementary Table 2 The DSP data after normalization to housekeeping proteins GAPDH and S6.

Supplementary Table 3 Overview of histopathological information for each patient and each ROI.
