# Supplementary material for: Digital spatial profiling identifies phospho-JNK as a biomarker for early risk stratification of aggressive prostate cancer
Source: Front Oncol. 2025 Jun 5;15:1572299. doi: 10.3389/fonc.2025.1572299 (PMC12176855; doi:10.3389/fonc.2025.1572299)
Supplement: Supplementary Table 1 — Overview of Nanostring GeoMx® DSP protein panels. [file Table1.docx]

**Supplementary Table 1**

| **Immune Cell Profiling** | **MAPK Signaling** | **Cell Death** | **PI3K/AKT signaling** |
| --- | --- | --- | --- |
| PD-1 | BRAF | BAD | Phospho-AKT1 (S473) |
| Pan-cytokeratin | EGFR | BCL6 | Phospho-GSK3B (S9) |
| CD68 | Phospho-JNK (T183/Y185) | BCLXL | Phospho-GSK3A (S21)/Phospho-GSK3B (S9) |
| HLA-DR | Phospho-MEK1 (S217/S221) | CD95/Fas | INPP4B |
| SMA | Phospho-p38 MAPK (T180/Y182) | GZMA | PLCG1 |
| Ki-67 | Phospho-p44/42 MAPK ERK1/2 (T202/Y204) | Cleaved Caspase 9 | Phospho-PRAS40 (T246) |
| Beta-2-microglobulin | pan-RAS | p53 | Phospho-Tuberin (T1462) |
| CD11c | Phospho-p44/42 MAPK ERK1/2 (T202/Y204) | PARP | Pan-AKT |
| CD20 | Phospho-p90 RSK (T359/S363) | BIM | MET |
| CD3 |  | Neurofibromin |  |
| CD4 |  |  |  |
| CD45 |  |  |  |
| CD56 |  |  |  |
| CD8 |  |  |  |
| CTLA4 |  |  |  |
| GZMB |  |  |  |
| PD-L1  Fibronectin |  |  |  |
| GAPDH |  |  |  |
| Histon H3 |  |  |  |
|  |  |  |  |
| S6 |  |  |  |
| Rb IgG |  |  |  |
| Ms IgG1 |  |  |  |
| Ms IgG2a |  |  |  |
